# Neoadjuvant durvalumab plus weekly nab-paclitaxel and dose-dense doxorubicin/cyclophosphamide in triple-negative breast cancer

**DOI:** 10.1038/s41523-021-00219-7

**Published:** 2021-02-08

**Authors:** Julia Foldi, Andrea Silber, Emily Reisenbichler, Kamaljeet Singh, Neal Fischbach, Justin Persico, Kerin Adelson, Anamika Katoch, Nina Horowitz, Donald Lannin, Anees Chagpar, Tristen Park, Michal Marczyk, Courtney Frederick, Trisha Burrello, Eiman Ibrahim, Tao Qing, Yalai Bai, Kim Blenman, David L. Rimm, Lajos Pusztai

**Affiliations:** 1 Section of Medical Oncology, Yale School of Medicine, New Haven, CT USA; 2 Department of Pathology, Yale School of Medicine, New Haven, CT USA; 3 Department of Surgery, Yale School of Medicine, New Haven, CT USA; 4 Department of Data Science and Engineering, Silesian University of Technology, Gliwice, Poland

**Keywords:** Breast cancer, Breast cancer

## Abstract

The goal of this Phase I/II trial is to assess the safety and efficacy of administering durvalumab concurrent with weekly nab-paclitaxel and dose-dense doxorubicin/cyclophosphamide (ddAC) neoadjuvant therapy for stages I–III triple-negative breast cancer. The primary endpoint is pathologic complete response (pCR:ypT0/is, ypN0). The response was correlated with PDL1 expression and stromal tumor-infiltrating lymphocytes (sTILs). Two dose levels of durvalumab (3 and 10 mg/kg) were assessed. PD-L1 was assessed using the SP263 antibody; ≥1% immune and tumor cell staining was considered positive; sTILs were calculated as the area occupied by mononuclear inflammatory cells over the total intratumoral stromal area. 59 patients were evaluable for toxicity and 55 for efficacy in the Phase II study (10 mg/kg dose). No dose-limiting toxicities were observed in Phase I. In Phase II, pCR rate was 44% (95% CI: 30–57%); 18 patients (31%) experienced grade 3/4 treatment-related adverse events (AE), most frequently neutropenia (*n* = 4) and anemia (*n* = 4). Immune-related grade 3/4 AEs included Guillain–Barre syndrome (*n* = 1), colitis (*n* = 2), and hyperglycemia (*n* = 2). Of the 50 evaluable patients for PD-L1, 31 (62%) were PD-L1 positive. pCR rates were 55% (95% CI: 0.38–0.71) and 32% (95% CI: 0.12–0.56) in the PD-L1 positive and negative groups (*p* = 0.15), respectively. sTIL counts were available on 52 patients and were significantly higher in the pCR group (*p* = 0.0167). Concomitant administration of durvalumab with sequential weekly nab-paclitaxel and ddAC neoadjuvant chemotherapy resulted in a pCR rate of 44%; pCR rates were higher in sTIL-high cancers.

## Introduction

The presence of immune cells in the tumor microenvironment of triple-negative breast cancer (TNBC) is associated with a good prognosis with^1^ or without adjuvant chemotherapy^2^, and is also predictive of pathologic complete response (pCR, ypT0/is, ypN0) after neoadjuvant chemotherapy^3^. Animal models of cancer also demonstrated that immune cells in the tumor microenvironment, particularly activated cytotoxic T cells, partially mediate chemotherapy response^4,5^. The availability of immune checkpoint inhibitors in the clinic that target the programmed cell death protein-1 (PD-1) and its ligands allow us to directly test if removing an important inhibitory signal from the immune microenvironment can lead to a more effective antitumor immune response and increase chemotherapy sensitivity. PD-1 is expressed on the surface of T cells and causes T-cell inhibition when it binds to either of its two ligands, programmed death-ligand-1 (PD-L1) and -2 (PD-L2). PD-L1 is expressed on the cell surface of cancer cells, macrophages, dendritic cells, and T cells^6,7^. There is a strong positive correlation between PD-L1 expression, immune infiltration, and tumor-infiltrating lymphocyte count, which explains the paradoxical observations that high PD-L1 expression is associated with better prognosis and higher pCR rate in breast cancer^8,9^.

Durvalumab is a monoclonal human immunoglobulin G1κ antibody that binds to PD-L1 and inhibits its interaction with PD-1 and CD80 (B7.1)^10^. The antibody also contains mutations in the constant domain of the heavy chain that reduces binding to complement protein C1q and to Fcγ receptors to avoid complement- and antibody-mediated cytotoxicity. In this trial (NCT02489448), we tested the hypothesis that durvalumab administered concurrently with sequential weekly nab-paclitaxel and dose-dense AC (ddAC) neoadjuvant chemotherapy will increase pCR rate above the historical pCR rate of 30% observed with the same chemotherapy regimen in TNBC in an earlier trial (SWOG S0800, NCT00856492)^11^. Among the different neoadjuvant chemotherapy options that can be combined with checkpoint inhibitors, we selected Nab-paclitaxel because it did not require steroid premedications^12^. In addition, there is preclinical evidence suggesting that nab-paclitaxel can release tumor antigens from rapidly dying cells^13^, which in turn might be able to prime antitumor T cells, a response that might be further amplified by the addition of checkpoint inhibitors.

## Results

### Patient population

Sixty-nine patients were screened for enrollment at Yale Cancer Center and its regional care centers; 60 patients consented to the trial between December 18, 2015, and November 21, 2018. One patient subsequently withdrew consent. The baseline characteristics of the remaining 59 patients are shown in Table 1. Seven patients were included in Phase I part of the study, four at 3 mg/kg and three at 10 mg/kg dose. Fifty-two patients were enrolled in the Phase II part at a 10 mg/kg dose. Two patients did not proceed to surgery—one developed irreversible altered mental status attributed to Guillen Barre syndrome and family opted for comfort care, the other completed treatment but died of sudden death in her home before undergoing surgery.

**Table 1:** Patients characteristics.

Characteristics	*N* (%)
All patients	59 (100)
*Age (median 50 years)*	
≤40	11 (19)
41–50	19 (32)
51–69	29 (49)
≥70	0 (0)
*Race/ethnicity*	
White (non-Hispanic)	35 (59)
Hispanic/Latino	5 (8)
Black	11 (19)
Asian/American Indian	4 (6)
Unknown	4 (6)
*Clinical tumor size*	
T1	21 (35)
T2	30 (51)
T3	8 (14)
*Clinical nodal status*	
cN0	31 (52)
cN1	25 (42)
cN2	1 (2)
cN3	3 (4)
*Clinical stage at diagnosis*	
I	12 (20)
II	33 (56)
III	14 (24)
*Histologic tumor grade*	
G1	1 (2)
G2	12 (20)
G3	45 (76)
Unknown	1 (2)
*Durvalumab dose level*	
3 mg/kg	4 (7)
10 mg/kg	55 (93)
*Pathologic response*	
pCR	26 (44)
RD	31 (53)
No surgery	2 (3)
*Residual cancer burden (RCB)*	
RCB-I	7 (12)
RCB-II	18 (31)
RCB-III	6 (10)
No surgery	2 (3)
*PD-L1 IHC*	
Negative	19 (32)
Positive (≥1%)	33 (56)
Not available	7 (12)
*Stromal TIL count*	
0–10%	28 (48)
11–29%	10 (17)
≥30%	16 (27)
Not available	5 (8)

*TIL* tumor-infiltrating lymphocytes, *IHC* immunohistochemistry.

### Efficacy

In the total intention-to-treat population in the Phase II trial who received the recommended Phase II dose of 10 mg/kg durvalumab (*N* = 55), the pCR rate was 44% (*N* = 24, 95% CI: 30–57%). Of the 55 patients, 19 (34.5%) received less than the planned 10 treatments with durvalumab, including 7 patients who received less than six doses. Among the 36 patients who received all 10 treatments, 17 (47%) had a pCR. Four patients had clinical progression and underwent surgery or switched to other chemotherapy. One of these patients who subsequently received carboplatin had a pCR at the surgery.

Among the 57 patients who received durvalumab at any dose level and completed surgery, pCR rate was 46%, including 2 pCRs among the 4 patients who received 3 mg/kg durvalumab in the phase I part of the study. The RCB class distribution was RCB-0 (pCR): 46%, RCB-I: 12%, RCB-II: 31%, and RCB-III: 10%. At a median follow-up of 20 months, there have been no recurrences in cases that achieved a pCR. Among those with residual disease, there were nine metastatic and two local recurrences. Three patients died from metastatic disease.

### Biomarker results

**Fig. 1:**
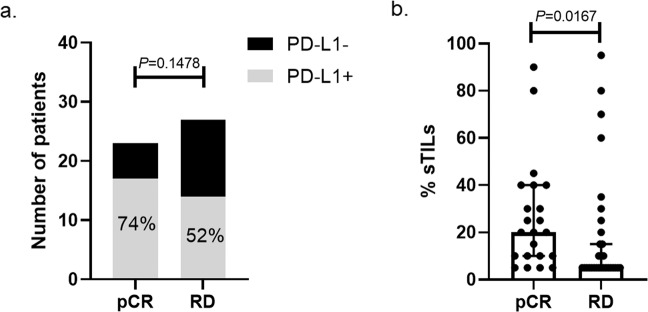
PD-L1 positivity and stromal TILs (sTILs) by pathologic response category. **a** PD-L1-positivity rate by SP263 antibody in the pathologic complete response (pCR, 74%, *n* = 14), and residual disease (RD, 52%, *n* = 13) groups, respectively, *p* = 0.148 (two-sided Fisher’s exact test). **b** The percentage of manual stromal tumor-infiltrating lymphocytes (sTILs) and the median (horizontal line) in the pCR (median: 20%) and RD groups (median: 5%), error bars represent 95% confidence intervals, *p* = 0.0167 (Mann–Whitney *U* test).

**Fig. 2:**
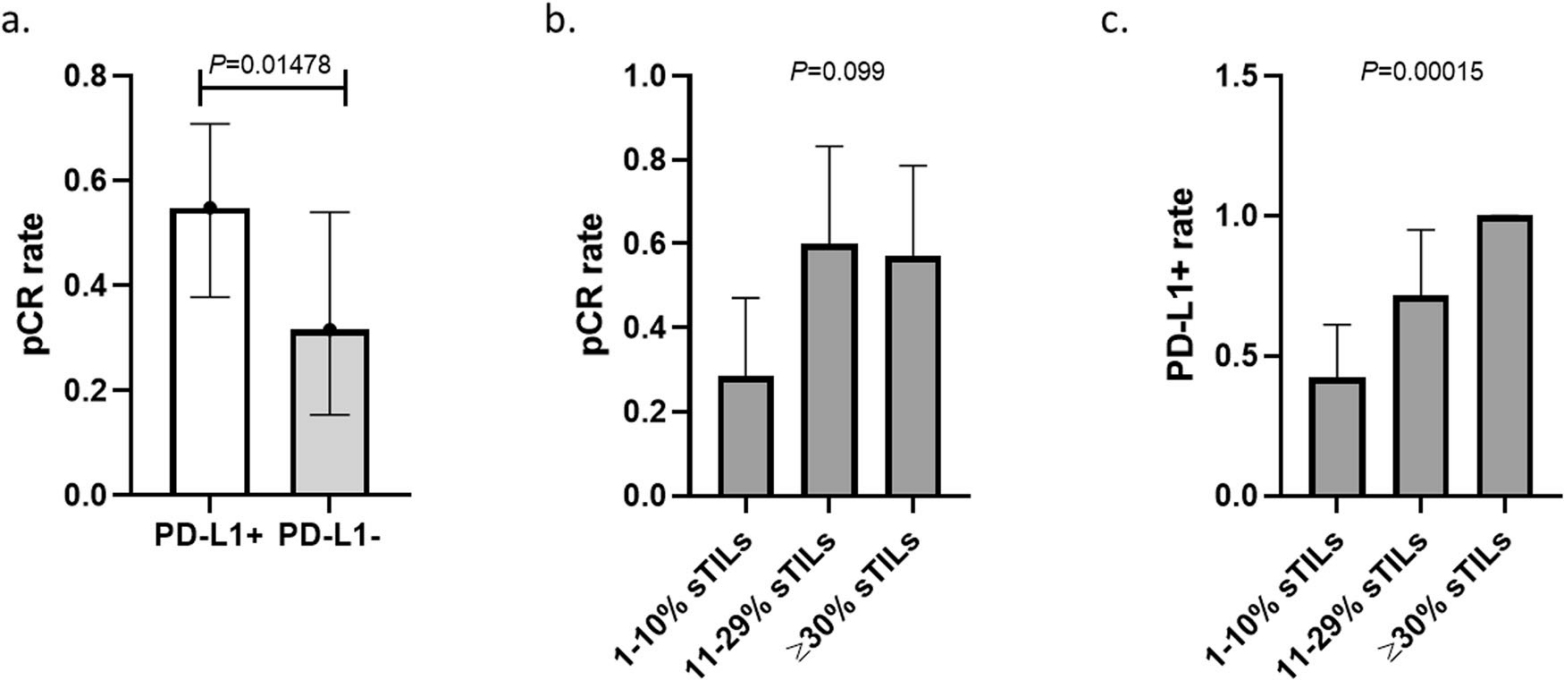
Distribution of pCR rate by PD-L1 status and manual stromal TILs (sTILs). **a** pCR rates in the PD-L1-positive (55%; 95% CI: 0.38–0.71) and -negative (32%; 95% CI: 0.15–0.54) groups, *p* = 0.148 (two-sided Fisher’s exact test). **b** pCR rates in cancers with ≥30% sTIL (pCR 57%), 29–10% sTIL (pCR 60%), and <10% sTIL (pCR 29%) groups, *p* = 0.099 (two-sided Fisher’s exact test). **c** PD-L1 positivity rates in cancers with sTIL >30%, sTIL 29%–10%, and sTIL <10%, were 100%, 71% and 42%, respectively, *p* = 0.00015 (two-sided Fisher’s exact test). On all panels error bars represent standard deviations (s.d.).

The consort diagram shows data availability for biomarker analysis (Supplementary Fig. 1). Fifty-two patients had PD-L1 IHC results available; 63% (*N* = 33) were PD-L1 positive. Two patients with PD-L1 staining had no surgery. Among the 50 patients who completed surgery, patients who achieved pCR had nominally higher PD-L1 positivity rate, compared to those with RD, although this did not reach not statistical significance (74% (95% CI: 54–88%) vs. 52% (95% CI: 34–88%); *p* = 0.15), (Fig. 1a). The pCR rate was 55% (95% CI: 38–71%) in the PD-L1 positive group compared to 32% (95% CI: 12–56%) in the PD-L1 negative group, also not significantly different (*p* = 0.15; Fig. 2a). In the same tissues, a parallel study with quantitative measurement of PD-L1 using immunofluorescence showed a statistically significant association between PD-L1 expression as a continuous variable and pCR, which is reported separately^14^.

Manual sTIL counts were available on 54 patients including the 2 patients who did not complete surgery. All patients with evaluable sTIL counts had at least 1% sTILs and 14 of the 52 patients (27%) had sTIL-high cancers defined as ≥30% sTILs. Figure 1b shows TIL counts in the pCR and RD groups. The pCR rates were 57%, 60%, and 29% among TIL-high (sTIL ≥ 30%), intermediate (sTIL 29–10%), and low (sTIL < 10%) groups, *p* = 0.099. (Fig. 2b). Forty-nine patients had both baseline sTIL and PD-L1 results available, Stromal TIL count was significantly higher in the PD-L1 positive group (median 27.5% vs. 5%; *p* < 0.001). In cancers with sTIL ≥30%, the PD-L1 positivity rate was 100%, in cancers with sTIL 11–29%, PD-L1 positivity was 71%, and in cancers with sTIL <10%, PD-L1 positivity was only 42% (*p* = 0.00015 (two-sided Fisher’s exact test); Fig. 2c). Patients whose tumors were PD-L1 positive and sTIL-high (≥30%) had a numerically higher pCR rate of 57% than those with PD-L1 positive but sTIL intermediate or low cancers (pCR rate 50%) or PD-L1 negative cancers (pCR rate 32%), but these differences did not reach statistical significance (*p* = 0.302, two-sided Fisher’s exact test).

In a multivariate analysis including PD-L1 expression (positive vs. negative), sTIL count (as a continuous variable), age, tumor size (T1 vs. T2/T3) NS nodal status (N− vs. N+), neither PD-L1 status (SP263 IHC) nor sTIL count was independently associated with pCR (Table 2).

**Table 2:** Univariate and multivariate logistic regression analyses to identify possible independent predictors of pCR following neoadjuvant therapy.

	Univariate analysis		Multivariate analysis^a^	
Variables	OR (95% CI)	*p*-value	OR (95% CI)	*p*-value
sTILs (continuous variable)	0.99 (0.98–1.01)	0.56	1.00 (0.98–1.01)	0.63
PD-L1 (POS vs. NEG)	2.63 (0.82–9.21)	0.11	2.62 (0.78–9.62)	0.13
Age (continuous variable)	1.00 (0.96–1.04)	0.94		
T status (T1 vs. T2/3)	0.47 (0.15–1.44)	0.19		
N status (N− vs. N+)	1.38 (0.49–4.00)	0.54		

^a^Covariates included are age (as a continuous variable), T status (T1 vs. T2/3), and N status (N− vs. N+).

### Safety and toxicity

All patients who received at least one dose of study-assigned therapy were evaluated for safety and toxicity. Overall, durvalumab was discontinued in 19 (32%) patients, including 2 patients in Phase I portion of the study. During nab-paclitaxel treatment durvalumab was discontinued in 9 patients, 5 due to local progression and 4 due to AEs, 3 of which were immune-related (irAE): one case each of autoimmune diabetes, Guillain–Barre syndrome (GBS), and optic neuritis. In addition, durvalumab was held in 3 patients for at least 1 cycle during nab-paclitaxel due to AEs—transaminitis, dermatitis, and fatigue—and was then continued with the AC portion of chemotherapy. During the AC portion of chemotherapy, durvalumab was discontinued in 9 patients, 7 due to AEs, 2 of which were irAEs: one pneumonitis and one dermatitis. In addition, 5 patients discontinued AC chemotherapy (one each for renal failure, fatigue, and rash, and two due to neutropenic fever) and proceeded to surgery before completing all the planned doses of durvalumab. Two patients were found to be ineligible for the AC portion of treatment due to underlying cardiac disease discovered during the study. Selected treatment-related and clinically relevant toxicities and immune-related adverse events reported within 180 days of the last investigational agent dose are summarized in Table 3. The irAEs observed have all previously been observed in the context of other clinical studies; no new safety concerns were identified. The most frequent irAEs reported were dermatitis and endocrinopathies, with thyroid dysfunction being the most common (hypo- and hyperthyroidism), occurring in 13% of patients including 4 patients who had hyperthyroidism that progressed to hypothyroidism. Adrenal insufficiency was observed in 1 patient. Two patients developed autoimmune diabetes characterized by low or undetectable C-peptide levels and in 1 of the 2 patients, autoantibodies against islet antigen 2 (IA-2).

**Table 3:** Treatment-related adverse events occurring in ≥10% of patients, or grades 3–4 occurring in ≥2% of patients.

	All grades	Grades 3–4
*Adverse event N (%)*		
Fatigue	50 (85)	1 (2)
Nausea	43 (73)	0 (0)
Alopecia	39 (66)	0 (0)
Anemia	35 (59)	4 (7)
Rash	35 (59)	1 (2)
Diarrhea	27 (46)	0 (0)
Peripheral sensory neuropathy	20 (37)	0 (0)
Leukopenia	16 (27)	3 (5)
Neutropenia	13 (22)	4 (7)
Vomiting	12 (20)	0 (0)
Anorexia	11 (19)	0 (0)
Dyspnea	9 (15)	1 (2)
Myalgia	9 (15)	0 (0)
Mucositis	7 (12)	1 (2)
ALT increased	7 (12)	0 (0)
Weight loss	7 (12)	0 (0)
Hypertension	6 (10)	0 (0)
Cough	6 (10)	0 (0)
Febrile neutropenia	3 (5)	3 (5)
Dehydration	3 (5)	2 (3)
*Immune-related adverse events*		
Hypothyroidism^a^	8 (13)	0 (0)
Hyperthyroidism^b^	4 (7)	0 (0)
Adrenal insufficiency	1 (2)	0 (0)
Diabetes mellitus	2 (3)	2 (3)
Dermatitis	12 (20)	0 (0)
Colitis	4 (7)	2 (3)
Guillan–Barre syndrome	1 (2)	1 (2)
Optic neuritis	1 (2)	0 (0)
Pneumonitis	1 (2)	0 (0)
Arthritis	1 (2)	0 (0)
Parotitis^c^	1 (2)	
SAE	14	n/a

*SAE* serious adverse event.

^a^Treatment-related adverse events were events that were attributed to a trial treatment by investigators.

^b^Four patients had both hyperthyroidism and hypothyroidism over the course of their treatment.

^c^Immune-related adverse event without a grade.

Two patients died. One discontinued therapy after one dose of durvalumab and two weekly treatments of nab-paclitaxel due to altered mental status attributed to Miller–Fisher variant of Guillain–Barre syndrome. Her mental status did not improve and the family opted for comfort care measures only. Her other co-morbid illnesses included hypertension, type 2 diabetes, and chronic obstructive pulmonary disease (COPD). The patient passed away several months later in a hospice. The other patient had completed 9 weekly treatments of nab-paclitaxel (further treatments were held because of peripheral neuropathy), 4 cycles of AC concurrent with durvalumab, and died of sudden death in her home before undergoing surgery. No autopsy was performed. Her other co-morbid illnesses included hypertension, hyperlipidemia, type 2 diabetes, history of coronary artery disease with a left ventricular ejection fraction (LVEF) of 50–55%.

## Discussion

The addition of ten cycles of durvalumab (10 mg/kg every 2 weeks) to weekly nab-paclitaxel (100 mg/m^2^) and ddAC resulted in a pCR rate of 44% (95% CI: 30–57%) in patients with early-stage TNBC in our trial. An identical chemotherapy regimen demonstrated a pCR rate of 29% in TNBC in the SWOG S0800 trial^11^, while other sequential taxane anthracycline regimens reported pCR rates between 30 and 48% in TNBC^15^. Two randomized Phase II trials also compared durvalumab plus chemotherapy with chemotherapy alone as neoadjuvant therapy. The GeparNuevo trial in TNBC demonstrated a numerical but not statistically significant increase in pCR rate (53% vs. 44%, *p* = 0.287) when durvalumab (1500 mg every 4 weeks) was included with weekly nab-paclitaxel (125 mg/m^2^) and epirubicin/cyclophosphamide^16^. The Bayesian randomized I-SPY2 trial evaluated the combination of 1500 mg durvalumab every 4 weeks and olaparib 100 mg twice a day concurrent with weekly paclitaxel (80 mg/kg) followed by AC without durvalumab or olaparib vs. the same chemotherapy regimen alone and reported an increase in pCR rate from 27 to 47% in the TNBC population of the trial with a 98% probability that the experimental arm is superior to the control^17^. The 95% confidence interval of the pCR point estimate in our trial includes the pCR rates seen in the immunotherapy arms of both these randomized trials and therefore the results are consistent with an improvement in pCR rate with the inclusion of durvalumab.

The addition of pembrolizumab to neoadjuvant chemotherapy was also examined in two large randomized trials in TNBC. The KEYNOTE-522 trial showed a significant improvement in pCR rate with the inclusion of pembrolizumab with paclitaxel plus carboplatin followed by anthracycline/cyclophosphamide compared to the same chemotherapy plus placebo (65% vs. 51%, *p* < 0.001)^18^. Another, previously reported arm of the I-SPY2 trial, randomized patients to 4 cycles of pembrolizumab vs. placebo in combination with weekly paclitaxel followed by AC without pembrolizumab and reported a significant improvement in predicted pCR rates from 22% in the control arm to 60% in the pembrolizumab arm in TNBC^19^. Atezolizumab has also been evaluated in two neoadjuvant randomized trials in TNBC. The IMpassion-031 trial randomized patients to atezolizumab or placebo concurrent with nab-paclitaxel followed by doxorubicin/cyclophosphamide, the same chemotherapy regimen as in our current study, and showed a significant increase in pCR rate (58% vs. 41%, *p* = 0.0044)^20^. However, one randomized trial, the NeoTRIPaPDL1, that compared nab-paclitaxel/carboplatin with or without atezolizumab failed to show a significant improvement in pCR rate with the inclusion of atezolizumab (pCR rate 43% vs. 41%)^21^. One important difference is that NeoTRIPaPDL1, unlike all the other positive trials had no anthracycline component. However, it is difficult to attribute the lack of efficacy in this trial to the lack anthracyclines; (i) it is clear from multiple metastatic trials in breast cancer that immune checkpoint inhibitors are synergistic with single-agent nab-paclitaxel at similar doses as used in NeoTRIPaPDL1, (ii) the two immune checkpoint inhibitor arms of the ISPY trial demonstrated improvement in pCR even though immunotherapy was only administered during the paclitaxel phase of chemotherapy, (iii) in lung cancer and other cancers immune checkpoint inhibitors are clearly synergistic with taxane/carboplatin regimens, and finally (iv) a small, multi-arm, window of opportunity trial, TONIC^22^, randomized patients to nivolumab alone or with a brief concurrent induction therapy including either irradiation (3 × 8 Gy), or cyclophosphamide (50 mg orally daily for 2 weeks), or cisplatin (40 mg/m^2^ intravenously weekly × 2), or doxorubicin (15 mg intravenously weekly × 2) for 2 weeks, and reported the highest responses rates and upregulation of immune-related genes with cisplatin and with doxorubicin. While it remains unclear why the NeoTRIPaPDL1 trial was negative, overall, the majority of trials provide consistent evidence for an improvement in pCR rate when an immune checkpoint inhibitor is added to standard of care neoadjuvant chemotherapy in TNBC.

Next, we examined the relationship between TIL count and pCR rate and found that patients with pCR had significantly higher TIL counts than those with residual disease. TIL-high (i.e., TIL ≥ 30%) cancers (*n* = 14) had a pCR rate close to 60%. However, TIL count alone may not identify patients who selectively benefit from the inclusion of an immune checkpoint inhibitor in the neoadjuvant chemotherapy regimen, as it has been shown that immune-rich TNBC also has higher pCR rates with chemotherapy alone compared to immune-low cancers^1–5^. The pCR rate was also higher in PD-L1 positive tumors (55% vs. 32%) in our study; however, this difference was not statistically significant (*p* = 0.15). The lack of statistical significance is likely due to the small sample size (i.e., the same proportions of 17/31 pCR in PD-L1 positive and 6/19 pCR in PD-L1 negative cancers would have resulted in a *p* < 0.0001 in a 500-patient trial [corresponding numbers would be 170/310 and 60/190]). All other substantially larger randomized neoadjuvant immune checkpoint inhibitor trials have reported significantly higher pCR rates in PD-L1 positive TNBC, which was consistent across three different immune checkpoint inhibitors—pembrolizumab^19^, durvalumab^16^, and atezolizumab^20,21^ and three different IHC assays: 22C3^19^, SP263^16^, and SP142^21^. However, these trials also showed that immune checkpoint therapy increases pCR rates even in PD-L1 negative cancers, and similar to TIL counts, PD-L1 status may not be useful in selecting patients for neoadjuvant immunotherapy.

The inability of PD-L1 protein expression, as determined by current assays, to identify patients who selectively benefit from immune checkpoint therapy in early-stage TNBC is very different from results obtained in metastatic TNBC where PD-L1 expression unequivocally identifies a subset of patients who have the potential to benefit from immune therapy. In the randomized IMpassion130 trial, only PD-L1 immune cell-positive patients (either with SP142, 22C3, or SP263 assays) showed improved progression-free survival (PFS) when atezolizumab was added to nab-paclitaxel as first-line therapy for metastatic TNBC^23^. In the KEYNOTE-119 trial, objective response rates and progression-free survival with single-agent pembrolizumab increased almost linearly as PD-L1 positivity increased (with 22C3 assay)^24^. The recently presented KEYNOTE 355 trial that compared pembrolizumab vs. placebo in addition to chemotherapy for metastatic TNBC in the first-line setting also demonstrated a statistically significant improvement in PFS in the pembrolizumab arm but only in PD-L1 positive cancers (CPS ≥ 10 using the 22C3 assay)^25^. The SAFIR-02 trial randomized patients with metastatic breast cancer who had a response or stable disease after 6 to 8 cycles of chemotherapy and had no actionable mutations, to maintenance single-agent durvalumab or continuation of chemotherapy. Maintenance durvalumab had inferior PFS in the entire population but demonstrated improved OS in the PD-L1 positive cancers (with SP142 assay)^26^. These results clearly demonstrate that unlike in stages I–III TNBC, PD-L1 positivity is required for the benefit of atezolizumab, pembrolizumab, and durvalumab in metastatic TNBC.

The biological reasons behind the distinct predictive functions of PD-L1 in metastatic vs. early-stage breast cancers are unclear. However, PD-L1 protein expression on immune cells, the primary cellular sources of PD-L1 expression in breast cancer, correlates closely with overall immune infiltration^6–8^ and metastatic lesions have been shown to have an overall more immune attenuated tissue microenvironment, even when immune cells are present, compared to primary tumors^27–29^. We hypothesize that in metastatic breast cancer, greater immune checkpoint inhibitor target expression (reflected by higher PD-L1 expression) may be required to obtain benefit from immune checkpoint inhibition, whereas low levels of the target (that may be missed by current PD-L1 IHC or TIL counting methods) may be sufficient to augment antitumor immune responses by immune checkpoint inhibitors in stages I–III TNBC. Indeed, in our study, all TNBCs had at least 1% sTILs but the PD-L1 positivity rate was only 42% in cancers with sTIL between 1 and 10%, compared to 100% positivity rate in cancers with sTIL ≥30%.

Immune-related adverse events were consistent with known adverse events of immune checkpoint inhibitors and no new safety concerns were identified. There were no perioperative complications. However, we did observe several severe irAEs including 2 patients (3%) with autoimmune type I diabetes. One patient presented with grade 3 hyperglycemia, diabetic ketoacidosis, low C-peptide, and increased islet antibody-2 (IA-2) after 4 cycles of durvalumab, requiring inpatient admission. She remains on long-term insulin treatment. The second patient presented with grade 4 hyperglycemia without diabetic ketoacidosis after completing 7 cycles of durvalumab. She had a history of metabolic syndrome (obesity, glucose intolerance) and was initially thought to have type II diabetes; however, her C-peptide level was low on presentation with a further decrease on follow-up testing 5 months later, indicating autoimmune type I-like diabetes. There were two deaths possibly related to treatment, one patient suffered a presumed cardiac arrest following completion of all study-related treatments but prior to undergoing surgery and one patient died after developing the Miller–Fisher variant of Guillain–Barre syndrome (GBS), a rare but previously reported neurologic complication of checkpoint inhibition characterized by ophthalmoplegia, ataxia, and hypersomnolence^30^. Notably, she developed GBS after only one dose of durvalumab. We recognize that our study had slightly higher than expected toxicity and mortality, we attribute this to more comorbidities in our study population than seen in the pivotal randomized trials. A growing number of randomized neoadjuvant trials with pembrolizumab, durvalumab, and atezolizumab including over 2000 patients with TNBC show good tolerability but also added immune-related toxicities. In the KEYNOTE-522 trial, 32% of patients experienced immune-related adverse events of any grade, and 12% had grade 3 or greater immune-related toxicities^18^. The most common were hypo-, and hyperthyroidism, and skin rash. Similar results were seen in Impassion-031^20^.

In summary, these results add to the growing literature that indicates the efficacy of immune checkpoint inhibitors in early-stage TNBC. Durvalumab concurrent with neoadjuvant nab-paclitaxel and ddAC chemotherapy resulted in a 44% pCR rate. Among the 62% of patients who had PD-L1 positive disease, the pCR rate was 55%, among PD-L1 negative cancers the pCR rate was 32%.

## Methods

### Study design

The primary objective of the Phase I part was to assess the safety of durvalumab concurrent with weekly nab-paclitaxel (100 mg/m^2^) × 12 treatments followed by doxorubicin (60 mg/m^2^) and cyclophosphamide (600 mg/m^2^) every 2 weeks (AC) × 4 treatments. Two dose levels, 3 mg/kg and 10 mg/kg, of durvalumab, administered every 2 weeks were explored following a 3 + 3 design. No steroid premedications were used during nab-paclitaxel treatment and durvalumab was administered immediately after completion of nab-paclitaxel. During AC, the first course of treatment was administered without dexamethasone pre-medication, but if clinically significant nausea or vomiting occurred subsequent courses were given with 20 mg dexamethasone. Approximately 24 h after administration of AC, 6 mg pegfilgrastim was administered followed by durvalumab. Dose-limiting toxicities (DLT) were monitored during the entire 20 weeks of therapy and for 4 weeks after completion of surgery before advancing to the next dose level. DLT was defined as any grade 4 immune-related adverse event (irAE), any grade 3 irAE that did not resolve to grade 2 within 3 days despite optimal management or did not resolve to ≤grade 1 within 14 days, and any ≥grade 3 non-irAE causally attributed to durvalumab. The primary efficacy objective was to assess pCR rate in patients who received the recommended Phase II dose including both the Phase I and phase II component. The efficacy study followed Simon’s two-stage design (*p*_0_ = 30%, *p*_1_ = 50%) with an interim efficacy analysis after the first 22 patients completed surgery and accrual was to be terminated if <7 patients experienced pCR, otherwise, accrual continued until 50 patients were evaluable for pCR. The maximum sample size was set to *N* = 61 allowing for replacement of non-evaluable patients. If >20 of 50 evaluable patients had pCR (40% observed pCR rate) the combination therapy would be recommended for further study. This was an investigator-initiated trial, and ethical approval was obtained from the Yale Human Investigations Committee (Yale University, HIC# 1409014537). Astra Zeneca provided study drug and funding for the trial but played no role in the study design, collection/analysis of data, or manuscript preparation.

### Patients and assessments

The study was approved and was annually reviewed by the internal institutional review board and all patients provided a written consent form to join the study. All patients signed written informed consent prior to participation. Patients with clinical stages I–III, triple-negative breast cancer, defined as ER and PR < 1% positive and HER2 negative (IHC 0, 1+, or 2+, or FISH negative), for whom systemic chemotherapy was indicated according to NCCN treatment guidelines were eligible^31^. Exclusion criteria included contraindications for anthracycline, paclitaxel, or anti-PD-L1 therapies (e.g., active autoimmune disease, live vaccines within 30 days, prior transplants, immune deficiency, active immunosuppressive medications).

Adverse events (AE) were assessed every 2 weeks and graded according to NCI CTCAE v4.03. All patients who received at least one dose of durvalumab were included in toxicity analysis. Surgery was performed within 4 weeks of completion of neoadjuvant chemotherapy and the extent of residual cancer assessed by the local pathologist as part of routine care. Residual Cancer Burden was assessed centrally by a breast pathologist (E.R.)^32^.

### Biomarker analysis

PD-L1 expression on formalin-fixed paraffin-embedded pretreatment biopsies was assessed with chromogenic immunohistochemistry (IHC) using the VENTANA PD-L1 (SP263) Assay performed according to the Federal Drug Administration (FDA) label. PD-L1 positivity was determined by consensus review of 2 pathologists (E.R., D.L.R.), and ≥1% staining on immune or tumor cells was considered positive. The percentage of stromal tumor-infiltrating lymphocytes (sTILs) was assessed on hematoxylin–eosin-stained slides and calculated as the area occupied by mononuclear inflammatory cells over the total intratumoral stromal area (E.R, K.S.). The association between pCR, PD-L1 expression, and sTILs along with clinicopathologic parameters (age, tumor size [T1 vs. T2/T3], nodal status [N0 vs. N1–N3]) was assessed using logistic regression. The pCR rates between PD-L1 positive and negative cohorts were compared using Fisher’s exact test. PD-L1 positivity rate in low, intermediate, and high sTIL cancers was assessed with a two-sided Fisher’s exact test. Median sTILs percent between cases with pCR and RD were compared using the Mann–Whitney *U* test.

### Reporting summary

Further information on research design is available in the Nature Research Reporting Summary linked to this article.

## Supplementary information

Supplementary Figure 1

Reporting Summary Checklist

## Data Availability

The data generated and analyzed during this study are described and shared openly in the following data record: 10.6084/m9.figshare.13362968^33^. The three data files containing all data are as follows. (1) Neoadjuvant Durvalumab Study_AEs_irAEs no dates.xlsx: Adverse events of all grades including detailed immune-related adverse events observed during our study. The file also includes data on the discontinuation of study drugs and the reason for those discontinuations. (2) Neoadjuvant Durvalumab Study_Demographics_Outcomes no dates.xlsx: Study participant demographics with no identifiable information and all dates removed from the data. Data includes baseline disease characteristics as well as outcomes in terms of survival and recurrence events up to the data cutoff of 8/15/2020. (3) Neoadjuvant Durvalumab Study_TIL counts_PDL1.xlsx - Tumor-infiltrating lymphocyte (TIL) counts (%) and PD-L1 status (positive vs. negative or unscorable) of patient’s tumors.
