# Author Correction: Neoadjuvant durvalumab plus weekly nab-paclitaxel and dose-dense doxorubicin/cyclophosphamide in triple-negative breast cancer

**DOI:** 10.1038/s41523-022-00392-3

**Published:** 2022-02-03

**Authors:** Julia Foldi, Andrea Silber, Emily Reisenbichler, Kamaljeet Singh, Neal Fischbach, Justin Persico, Kerin Adelson, Anamika Katoch, Nina Horowitz, Donald Lannin, Anees Chagpar, Tristen Park, Michal Marczyk, Courtney Frederick, Trisha Burrello, Eiman Ibrahim, Tao Qing, Yalai Bai, Kim Blenman, David L. Rimm, Lajos Pusztai

**Affiliations:** 1 Section of Medical Oncology, Yale School of Medicine, New Haven, CT USA; 2 Department of Pathology, Yale School of Medicine, New Haven, CT USA; 3 Department of Surgery, Yale School of Medicine, New Haven, CT USA; 4 Department of Data Science and Engineering, Silesian University of Technology, Gliwice, Poland

**Keywords:** Breast cancer, Breast cancer

Correction to: *npj Breast Cancer* 10.1038/s41523-021-00219-7, published online 8 February 2021

The original version of the published Article had an error in the Author affiliations. The second affiliation for co-Author Michal Marczyk was inadvertently omitted. The following affiliation has been added: Department of Data Science and Engineering, Silesian University of Technology, Gliwice, Poland. The HTML and PDF versions of the Article have been corrected.

