# Supplementary figures and images for: Neoadjuvant durvalumab plus weekly nab-paclitaxel and dose-dense doxorubicin/cyclophosphamide in triple-negative breast cancer
Source: NPJ Breast Cancer. 2021 Feb 8;7:9. doi: 10.1038/s41523-021-00219-7 (PMC7870853; doi:10.1038/s41523-021-00219-7)

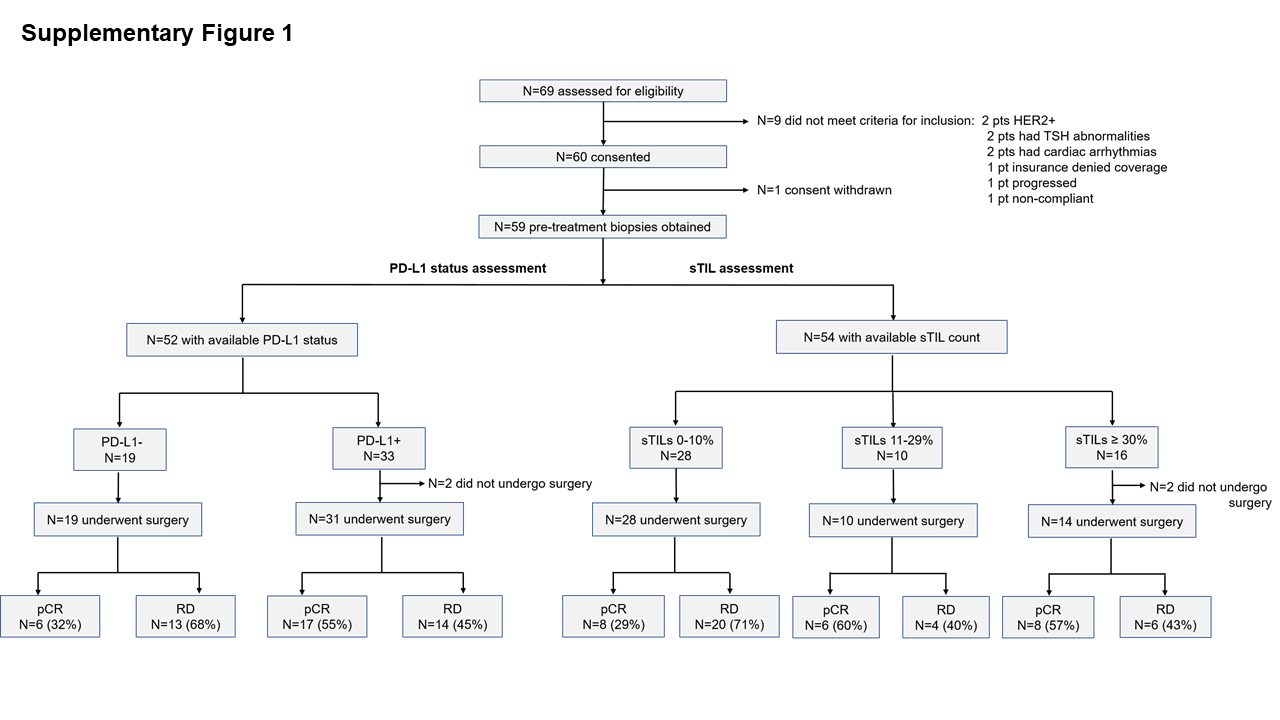

Supplement: Supplementary file 1 — Supplementary Figure 1 [file 41523_2021_219_MOESM1_ESM.tif]
