# Supplementary material for: Neoadjuvant durvalumab plus weekly nab-paclitaxel and dose-dense doxorubicin/cyclophosphamide in triple-negative breast cancer
Source: NPJ Breast Cancer. 2021 Feb 8;7:9. doi: 10.1038/s41523-021-00219-7 (PMC7870853; doi:10.1038/s41523-021-00219-7)
Supplement: Supplementary file 2 — Reporting Summary Checklist [file 41523_2021_219_MOESM2_ESM.pdf]

nature research

Corresponding author(s): Lajos Pusztai, MD DPhil

Last updated by author(s): Dec 17, 2020

Reporting Summary

Nature Research wishes to improve the reproducibility of the work that we publish. This form provides structure for consistency and transparency in reporting. For further information on Nature Research policies, see our Editorial Policies and the Editorial Policy Checklist.

Statistics

For all statistical analyses, confirm that the following items are present in the figure legend, table legend, main text, or Methods section.

n/a	Confirmed	
☐	☒	The exact sample size (n) for each experimental group/condition, given as a discrete number and unit of measurement
☐	☒	A statement on whether measurements were taken from distinct samples or whether the same sample was measured repeatedly
☐	☒	The statistical test(s) used AND whether they are one- or two-sided<br>*Only common tests should be described solely by name; describe more complex techniques in the Methods section.*
☐	☒	A description of all covariates tested
☐	☒	A description of any assumptions or corrections, such as tests of normality and adjustment for multiple comparisons
☐	☒	A full description of the statistical parameters including central tendency (e.g. means) or other basic estimates (e.g. regression coefficient) AND variation (e.g. standard deviation) or associated estimates of uncertainty (e.g. confidence intervals)
☐	☒	For null hypothesis testing, the test statistic (e.g. F, t, r) with confidence intervals, effect sizes, degrees of freedom and P value noted<br>*Give P values as exact values whenever suitable.*
☒	☐	For Bayesian analysis, information on the choice of priors and Markov chain Monte Carlo settings
☒	☐	For hierarchical and complex designs, identification of the appropriate level for tests and full reporting of outcomes
☒	☐	Estimates of effect sizes (e.g. Cohen's d, Pearson's r), indicating how they were calculated

Our web collection on statistics for biologists contains articles on many of the points above.

Software and code

Policy information about availability of computer code

Data collection	No software was used. Data collected from patients treated at a US academic institution on a clinical trial was used.
Data analysis	GraphPad Prism Version 8.4.2. and R Studio Version 3.6.2 were used for data analysis

For manuscripts utilizing custom algorithms or software that are central to the research but not yet described in published literature, software must be made available to editors and reviewers. We strongly encourage code deposition in a community repository (e.g. GitHub). See the Nature Research guidelines for submitting code & software for further information.

Data

Policy information about availability of data

All manuscripts must include a data availability statement. This statement should provide the following information, where applicable:
- Accession codes, unique identifiers, or web links for publicly available datasets
- A list of figures that have associated raw data
- A description of any restrictions on data availability

The dataset used and/or analyzed during the current study are available from the corresponding author on reasonable request and approval from the study sponsor according to available guidelines at the time of the request.

Field-specific reporting

Please select the one below that is the best fit for your research. If you are not sure, read the appropriate sections before making your selection.

☒ Life sciences ☐ Behavioural & social sciences ☐ Ecological, evolutionary & environmental sciences

For a reference copy of the document with all sections, see nature.com/documents/nr-reporting-summary-flat.pdf

Life sciences study design

All studies must disclose on these points even when the disclosure is negative.

Sample size	The sample size was determined by the 3+3 and Simons two stage designs. During the Phase I portion of the trial, a minimum of 6 and a maximum of 12 patients were planned to be enrolled. The efficacy study followed Simon's two stage design (p0=30%, p1=50%) with an interim efficacy analysis after the first 22 patients completed surgery and accrual was to be terminated if < 7 patients experienced pCR, otherwise accrual continued until 50 patients were evaluable for pCR. Maximum sample size was set to N=61 allowing for replacement of non-evaluable patients. If >20 of 50 evaluable patients had pCR (40% observed pCR rate) the combination therapy would be recommended for further study.
Data exclusions	Clinical trial exclusion criteria included contraindications for anthracycline, paclitaxel or anti-PD-L1 therapies (e.g. active autoimmune disease, live vaccines within 30 days, prior transplants, immune deficiency, active immunosuppressive medications).<br>All patients who received the recommended Phase II dose of 10 mg/kg durvalumab were included in the efficacy analysis.<br>All patients who received at least one dose of durvalumab were included in toxicity analysis.<br>Patients whose tumors did not have available TIL counts or PD-L1 expression recorded data were excluded from the biomarker analysis.
Replication	All experimental findings are reproducible.
Randomization	This was a single arm study.
Blinding	This was an open label unblinded study.

Reporting for specific materials, systems and methods

We require information from authors about some types of materials, experimental systems and methods used in many studies. Here, indicate whether each material, system or method listed is relevant to your study. If you are not sure if a list item applies to your research, read the appropriate section before selecting a response.

Materials & experimental systems

n/a	Involved in the study	
☐	☒	Antibodies
☒	☐	Eukaryotic cell lines
☒	☐	Palaeontology and archaeology
☒	☐	Animals and other organisms
☐	☒	Human research participants
☐	☒	Clinical data
☒	☐	Dual use research of concern

Methods

n/a	Involved in the study	
☒	☐	ChIP-seq
☒	☐	Flow cytometry
☒	☐	MRI-based neuroimaging

Antibodies

Antibodies used	VENTANA PD-L1 (SP263) Assay
Validation	VENTANA PD-L1 predictive assays identify patients who are most likely to respond to specific therapies, including durvalumab and is FDA-approved as a predictive test in urothelial carcinoma.

Human research participants

Policy information about studies involving human research participants

Population characteristics	Eligible participants were patients with clinically stage I-III, triple negative breast cancer, defined as ER and PR<1% positive and HER2 negative (IHC 0, 1+ or 2+, or FISH negative), for whom systemic chemotherapy was indicated according to NCCN treatment guidelines were eligible 31. Exclusion criteria included contraindications for anthracycline, paclitaxel or anti-PD-L1 therapies (e.g. active autoimmune disease, live vaccines within 30 days, prior transplants, immune deficiency, active immunosuppressive medications).

Recruitment	All eligible patients in this study were treated at a US academic oncology clinic.
Ethics oversight	This was an investigator-initiated trial, and ethical approval was obtained from the Yale Human Investigations Committee (Yale University, HIC# 1409014537).

Note that full information on the approval of the study protocol must also be provided in the manuscript.

Clinical data

Policy information about clinical studies

All manuscripts should comply with the ICMJE guidelines for publication of clinical research and a completed CONSORT checklist must be included with all submissions.

Clinical trial registration	NCT02489448
Study protocol	Available as a supplementary document with this manuscript.
Data collection	Data were collected between 12/28/2015 and 5/20/2019 from the Yale Cancer Center/Smilow Cancer Hospital treating patients fitting the eligibility criteria for the study.
Outcomes	The primary objective of the Phase I portion of the trial was to assess the safety of MEDI4736 combined with chemotherapy and determine if full dose of durvalumab can be administered concomitantly with full dose weekly nab-paclitaxel followed by dose-dense AC chemotherapies, respectively.<br>The primary objective of the Phase II portion of the study was to estimate the pCR rate with durvalumab in combination with weekly nab-paclitaxel x 12 treatments followed by durvalumab in combination with ddAC x 4 treatments for estrogen receptor (ER), progesterone receptor (PR) and HER2 negative (triple negative, TNBC), clinical stage I-III breast cancer. Pathologic complete response is defined as the absence of residual invasive cancer on hematoxylin and eosin evaluation of the resected breast specimen and all sampled regional lymph nodes following completion of neoadjuvant systemic therapy (i.e. ypT0/Tis ypN0).<br>Secondary objectives were to assess the safety and toxicity of adding anti-PD-L1 antibody, durvalumab to standard of care neoadjuvant chemotherapy in the Phase II portion of the trial. Safety was be assessed by using Common Terminology Criteria for Adverse Events, Version 4.3. We also monitored for events of special clinical interest with a suspected auto-immunologic etiology including grade ≥3 colitis, hyperthyroidism, hypophysitis, hypothyroidism, pneumonitis, rash and anti-drug-antibody (ADA) immune complex disease (manifested by symptoms of arthralgias, abdominal pain, back pain, and vasculitis). For patients included in the Phase II portion of the trial, toxicity was be reported separately for acute toxicities observed on-therapy and for delayed toxicities observed during a 90-day follow up period after completion of therapy. Acute toxicities were also reported separately for the combination with nab-paclitaxel and dose dense AC parts of the treatment.
